# Use of the Er,Cr:YSGG laser as an adjuvant in root canal system disinfection

**DOI:** 10.4317/jced.62149

**Published:** 2024-11-01

**Authors:** Idalia Rodríguez-Delgado, Daniela Hernandez-Martinez, Isaac Kably-Mizrahi, Marco Antonio Garza-Navarro, Jorge Jaime Flores-Treviño, Elizabeth Madla-Cruz, Myriam Angélica De la Garza-Ramos

**Affiliations:** 1 Universidad Autónoma de Nuevo León. Facultad de Odontología, Posgrado de Endodoncia. San Nicolás de los Garza, Nuevo León, México; 2 Universidad Autónoma de Nuevo León, Facultad de Ingeniería Mecánica y Eléctrica, San Nicolás de los Garza, Nuevo León, México; 3 Universidad Autónoma de Nuevo León. Facultad de Odontología, Departamento de Microbiología Oral. San Nicolás de los Garza, Nuevo León, México

## Abstract

**Background:**

This study investigated the antibacterial efficacy and cleaning capacity of the Er,Cr:YSGG laser in root canal disinfection.

**Material and Methods:**

Fifteen teeth were inoculated with an *Enterococcus faecalis* biofilm and assigned to three groups as follows: Group 1 (control: biofilm only), Group 2 (Ultrasound [US] + 5.25% NaOCl), and Group 3 (Er, Cr at 1.25W, 40 Hz, with a steriline saline water-air spray 30-90%). Samples were taken from the root canals, and two from each group were prepared for a permeability experiment, where 2% methylene blue was placed inside the conduits and then incubated at 37°C for 20 min. The roots were sectioned into cervical, middle, and apical thirds and viewed with an optical microscope to observe dye penetration. The remaining samples (n=3) were prepared for SEM to analyze dentinal tubule permeability. Optical density was analyzed before and after the treatments. Normality was assessed using the Shapiro-Wilk test, and data were analyzed using Student’s t-test and ANOVA with a significance level of 5%.

**Results:**

Group 2 had a greater colony forming unit reduction with no difference from Group 3. Group 3 had greater methylene blue penetration and dental tubule cleaning. Both methods are effective in bacterial reduction; however, the reduction was greater with 5.25% NaOCl and ultrasound.

**Conclusions:**

Er,Cr:YSGG laser significantly removes the smear layer.

** Key words:**Er, Cr: YSGG, Ultrasound, Dentinal tubules, SEM, E. faecalis.

## Introduction

Effective disinfection of root canals is critical to the success of endodontic treatment, achievable only by eliminating dentinal debris and microorganisms (1). Endodontic treatment success rates are reported between 85% and 90%, with the primary cause of failure being persistent microorganisms (2). Sodium hypochlorite (NaOCl) solutions, used at concentrations between 2.5% and 5%, are potent disinfectants with tissue solubility and proteolytic properties against microorganisms (3). However, its antibacterial efficacy and tissue dissolution capacity are concentration-dependent and proportionally related to toxicity (4). The dentinal tubules in tooth and root structures offer refuge for microorganisms, complicating disinfection efforts (5).

Therefore, techniques such as passive ultrasound irrigation and laser-activated irrigation have been developed to enhance irrigant dispersion within the root canal system. Passive ultrasound irrigation transmits acoustic energy from an oscillating tip into the irrigation solution within the root canal (6). Alternatively, laser-activated irrigation, utilizing the erbium, chromium: yttrium scandium gallium garnet laser (Er:YAG: 2980 nm–Er,Cr:YSGG: 2780 nm) with a 2,790 nm wavelength, delivers energy through flexible fiber optic tips. This laser’s high absorption in steriline saline water and affinity for hydroxyapatite makes it suiTable for root canal treatment (7).

Erbium laser absorption in steriline saline water initially causes thermal effects, creating vapor bubbles that generate microjets, producing high shear stresses along the dentin walls, effectively removing debris and biofilm layers (8). Therefore, we investigated the antibacterial efficacy and cleaning capacity of the Er,Cr laser as a root canal system disinfection. It is anticipated that the results will be utilized to enhance clinical endodontic treatments and improve their success rates, leading to a significant increase in treatment efficacy.

## Material and Methods

-Phase I

1. Pilot Test for Biofilm Training

The sample size for this study was determined based on existing literature and anticipated effect sizes. While a formal power analysis was not conducted prior to data collection, the sample was carefully selected to ensure adequate representation and minimize the risk of Type II errors. Therefore, fourteen teeth were sectioned 1 mm below the cementoenamel junction (CEJ), and the clinical crowns were discarded. Uniform dentin blocks measuring 4 mm in length and 2 mm in width were obtained. Samples were cleaned with ultrasound in 2.5% NaOCl and EDTA for 2 minutes and rinsed with normal saline to inhibit EDTA effects.

2. Sterilization of Dentin Samples

Dentin blocks were sterilized in a hermetically sealed glass jar in an autoclave at 121°C for 15 minutes. To ensure an aseptic environment during the collection of biofilm samples, several stringent protocols were followed. First, the sampling area was disinfected with (specific disinfectant) to minimize surface contamination. All instruments used were sterilized prior to the procedure, and sterile gloves and masks were worn by the personnel involved. The biofilm samples were collected using sterile tools, and immediately placed in sterile containers to prevent any exposure to external contaminants. Additionally, the collection process was conducted in a controlled environment with limited access to reduce the risk of airborne contamination.

3. Biofilm Formation with *Enterococcus faecalis* in Dentin Blocks

*E. faecalis* strains were activated in brain heart infusion (BHI) following ATCC specifications to prepare the biofilm. A culture containing 1.4 mL of 10^6 cells/mL was incubated to the exponential phase. Dentin blocks were submerged in 1 mL of sterile brain heart infusion broth and incubated in a laminar flow chamber for 7 days at 37°C, with the broth replaced every 48 hours. The experiment followed Zhu *et al*. (9) methodology, hence dentin blocks were washed with phosphate-buffered saline under pressure, immersed in Eppendorf tubes, and activated by vortexing for one minute, repeated three times to remove loosely adhered bacteria. Biofilm viability was confirmed by optical density testing.

-Phase II

1. Sample Selection

Fifteen extracted single-root human teeth from adults aged between 18 and 65 years were included in the study, provided that positive informed consent was obtained. The study focused on adult molars with infections, including abscesses, sinus tracts, or signs of irreversible pulpitis. Radiographically, eligible teeth were those exhibiting interradicular or periapical radiolucency and less than two-thirds root resorption. Teeth were excluded if they had abnormal anatomy, calcified canals, unrestorable crowns, or evidence of extensive internal or external pathological root resorption. Additionally, patients with any systemic disorders or medically compromising conditions, or those who had received antibiotics within two weeks prior to microbial sampling, were excluded from the study. Teeth were preserved in saline solution post-extraction.

2. Specimen Preparation

Teeth were marked at the CEJ and the clinical crowns were removed using a low-speed handpiece (KaVo Model: EXPERTmatic C/A Low Speed H/P Lux 1:1 E20L Country of Manufacture: Germany) with a diamond disc, resulting in root lengths of 14 mm. The canal patency was checked using a #10 K-type file, and the working length was established 1 mm short of the clinical apical foramen. The Wave One Gold instrumentation system (Dentsply Sirona®) was employed with an X-Smart Plus® motor to prepare the root canals to a master apical file size of 45/05. The canals were irrigated with 5.25% NaOCl during each instrumentation.

3. Teeth Sterilization

Prepared canals were placed in a hermetically sealed glass jar and sterilized in an autoclave at 121°C for 15 minutes.

-Phase III

1. Biofilm Formation with *E. faecalis* in the Root Canals

Following the pilot study, *E. faecalis* was introduced into the root canals for biofilm formation. Samples were divided into three groups: Group 1 (control: biofilm only), Group 2 (Ultrasound (US) + 5.25% NaOCl), and Group 3 (Er, Cr at 1.25W, 40 Hz, with a steriline saline water-air spray 30-90%).

2. Microbiological Sample Collection Before Treatment

Before treatment, biofilm samples were collected from the root canals under aseptic conditions. A sterile paper tip was rubbed against the canal walls and placed in an Eppendorf tube containing 1,000 μL of trypticasein soy broth. After 24 hours of incubation, serial dilutions were performed, and the samples were seeded on trypticasein soy agar plates and incubated for another 24 hours. Bacterial growth was quantified using optical density measurements.

-Phase IV

1. Ultrasound Protocol

The ultrasound device used in this study was the EightTeeth Ultrasonic Device, Model 9®. This device operates at a frequency of 25 kHz and is equipped with a #20 ultrasonic tip. It provides high-efficiency cleaning through its cavitation and acoustic streaming capabilities. The choice of this specific model was based on its proven effectiveness in enhancing irrigant dispersion and its suitability for endodontic applications as demonstrated in previous studies. Group 2, consisting of 5 teeth, was irrigated with 5.25% NaOCl and activated with a #20 US tip introduced up to 2 mm short of the apex at 45 kHz. Activation was conducted for 15 seconds, followed by a 30-second cooling period, repeated three times.

2. Er, Cr Laser Protocol

Group 3, also consisting of 5 teeth, was irradiated with an Er, Cr laser using a 200-micron tip introduced up to 2 mm short of the apex. The laser tip was sterilized between each sample to ensure that no cross-contamination occurred throughout the procedure. The sterilization process involved the use of an autoclave, where the laser tip was placed in a sterilization pouch and subjected to high-pressure steam, ensuring complete sterilization. This method guarantees that the tip was free from any biological contaminants before use on a new sample. Alternatively, chemical sterilization with an approved disinfectant was utilized in cases where autoclaving was not feasible, adhering to standard infection control protocols.

Parameters were set to 1.25W, 40 Hz, steriline saline water-air spray at 90-30%, and activation speed of 2 mm/s for 15 seconds, with a 30-second cooling period, repeated three times. During the application of the Er,Cr laser, a steriline saline water spray was used to cool the treatment area and facilitate the removal of debris. The steriline saline water spray, delivered simultaneously with the laser energy, helps to maintain the temperature of the tissue within a safe range, reducing the risk of thermal damage. No additional solutions were applied during the procedure, ensuring that the laser’s effects were precise and localized.

3. Microbiological Sample Collection After Treatment

Post-treatment root canal samples were collected from both groups using the same methodology as before treatment.

-Phase V

1. Sample Preparation for the Permeability Experiment

Two samples from each group were prepared for permeability testing. The tooth vertices were sealed with sticky wax, and the roots were sealed externally with three layers of ethyl cyanoacrylate, dried, and transferred individually to Eppendorf tubes. Samples were filled with 2% methylene blue dye using a hypodermic syringe and incubated at 37°C for 20 minutes, then rinsed thoroughly with running steriline saline water. The root canals were dried with absorbent paper tips. The first 2 mm of the neck were excluded from the study, and the remaining root was sectioned horizontally into three segments representing the cervical, middle, and apical thirds. Sections were washed with distilled steriline saline water and observed under an optical microscope.

2. Evaluation of Irrigant Penetration by Optical Microscopy

Segments were analyzed using a ZEISS® optical microscope at 20x magnification. Images were exported and analyzed with ImageJ software to quantify methylene blue penetration.

3. Evaluation of Tubular Permeability by Scanning Electron Microscope

Samples were dried completely, mounted on metal slides for SEM, marked to divide them into cervical, middle, and apical thirds, placed in a centrifuge to create a vacuum, and measurements were taken. SEM images of the cervical, middle, and apical surfaces at 6000x and 12000x magnifications were selected to visualize the surface and dentinal tubules. Images were qualitatively evaluated by endodontists in a single-blind manner. Normality was assessed using the Shapiro-Wilk test, and data were analyzed using Student’s t-test and ANOVA with a significance level of 5%.

## Results

**Figure 1:**
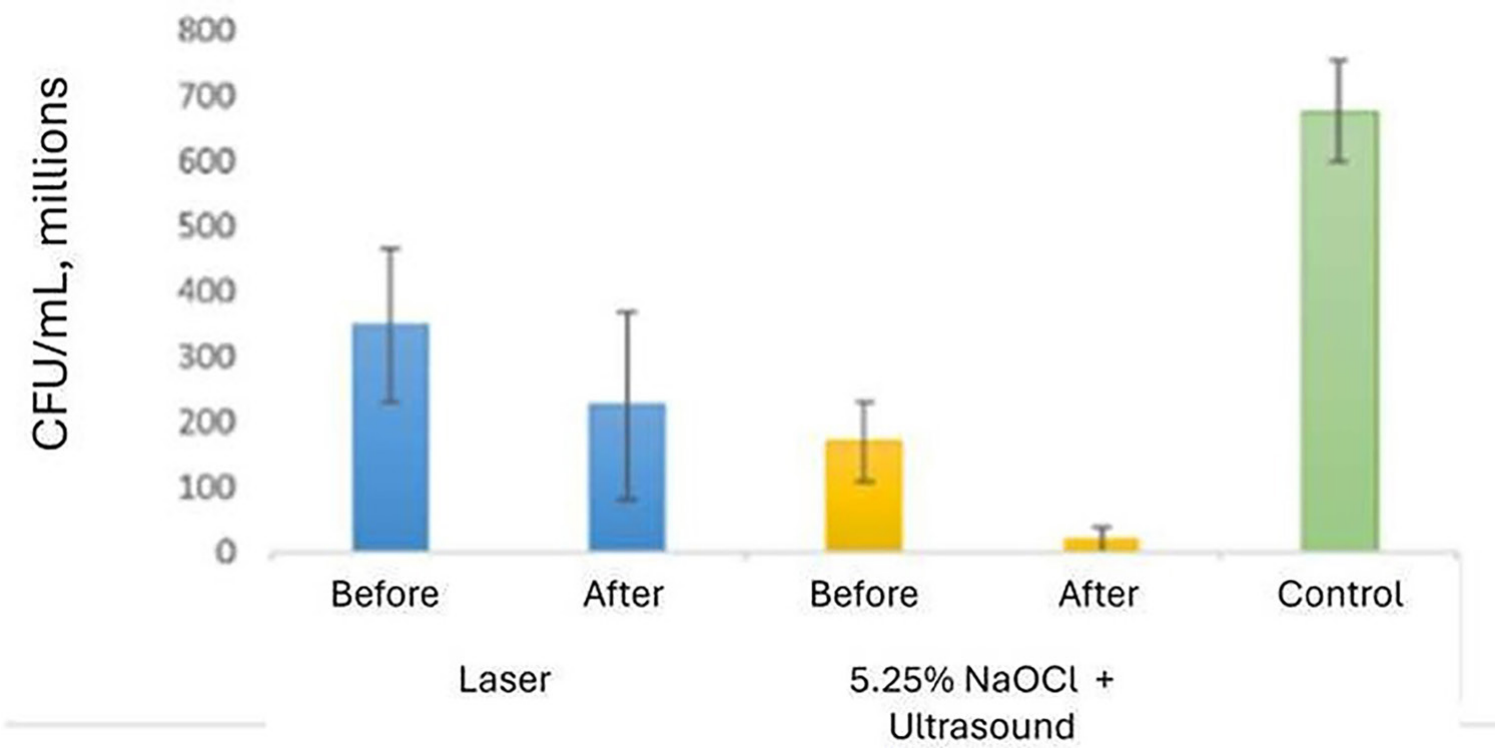
Difference in the mean bacterial growth before and after treatments.

**Figure 2:**
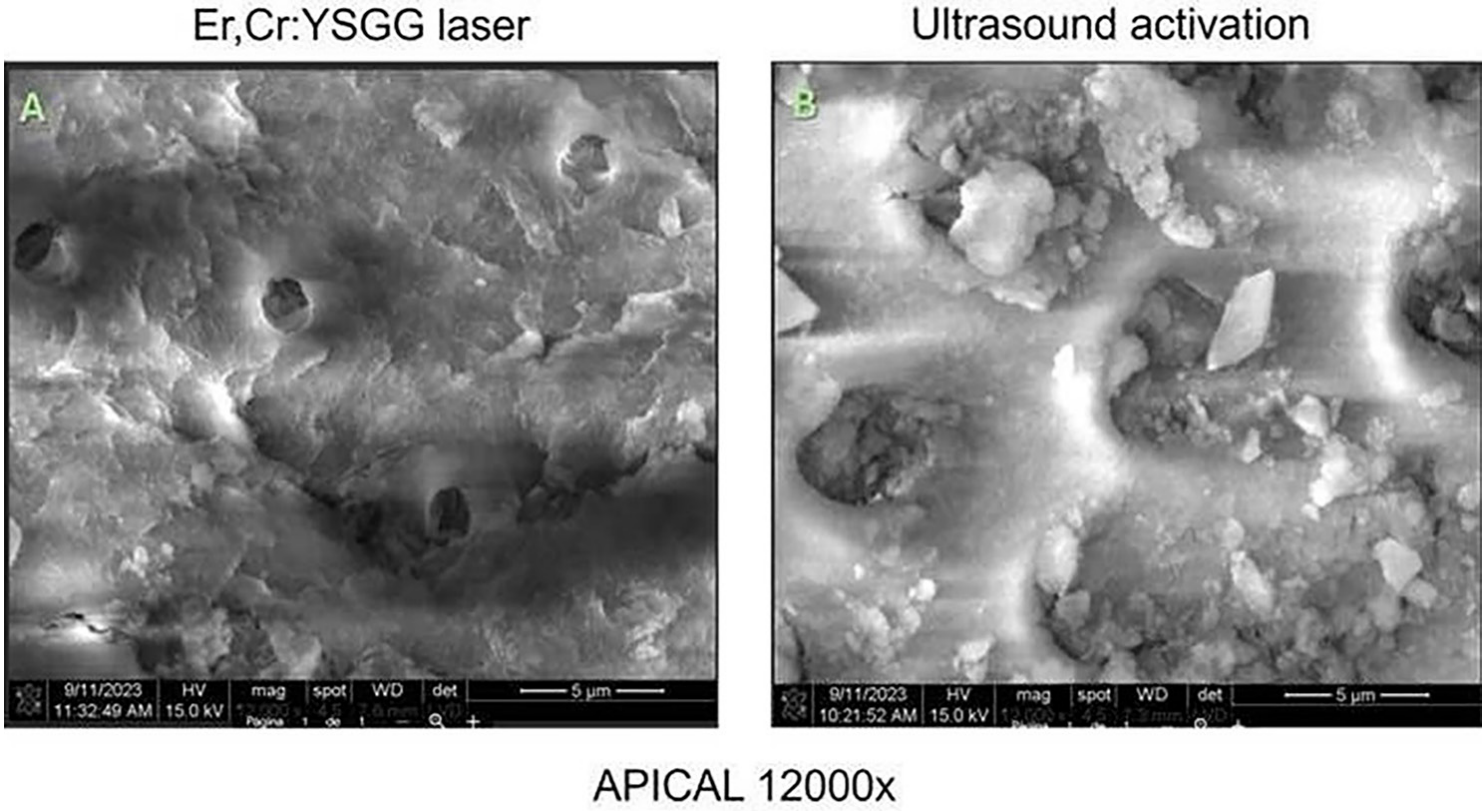
A. Few permeable dentinal tubules are observed, the rest of the surface is seen with a layer of dentinal sludge (semar layer). 2 B. Dentinal tubules with dentinal sludge (semar layer) are observed.

**Figure 3:**
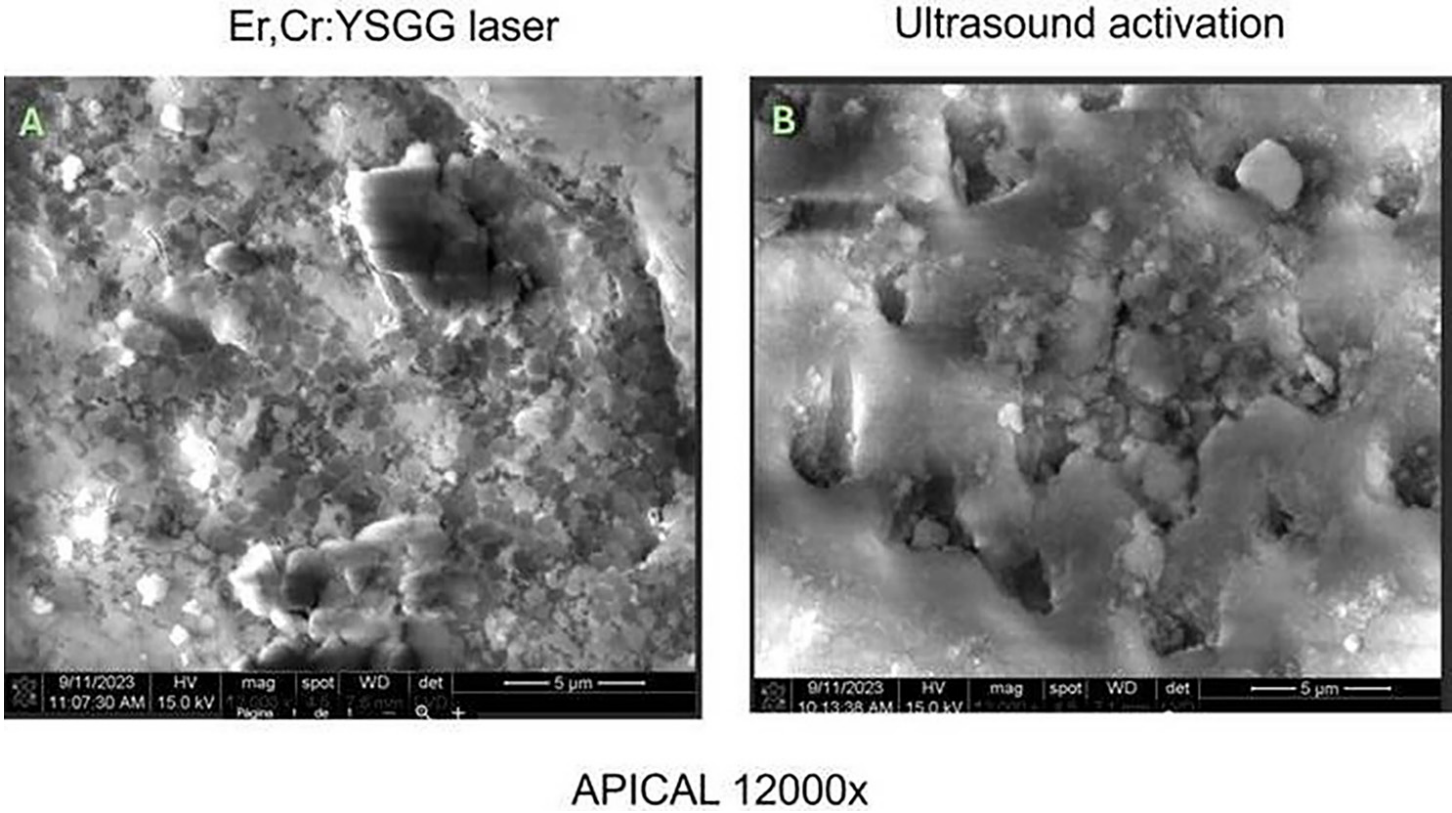
A. Dentinal tubules are not visible; dentinal sludge is seen covering the entire tooth surface with cocci. 3 B. Nonpermeable dentinal tubules are observed with a light layer of dentinal sludge.

**Figure 4:**
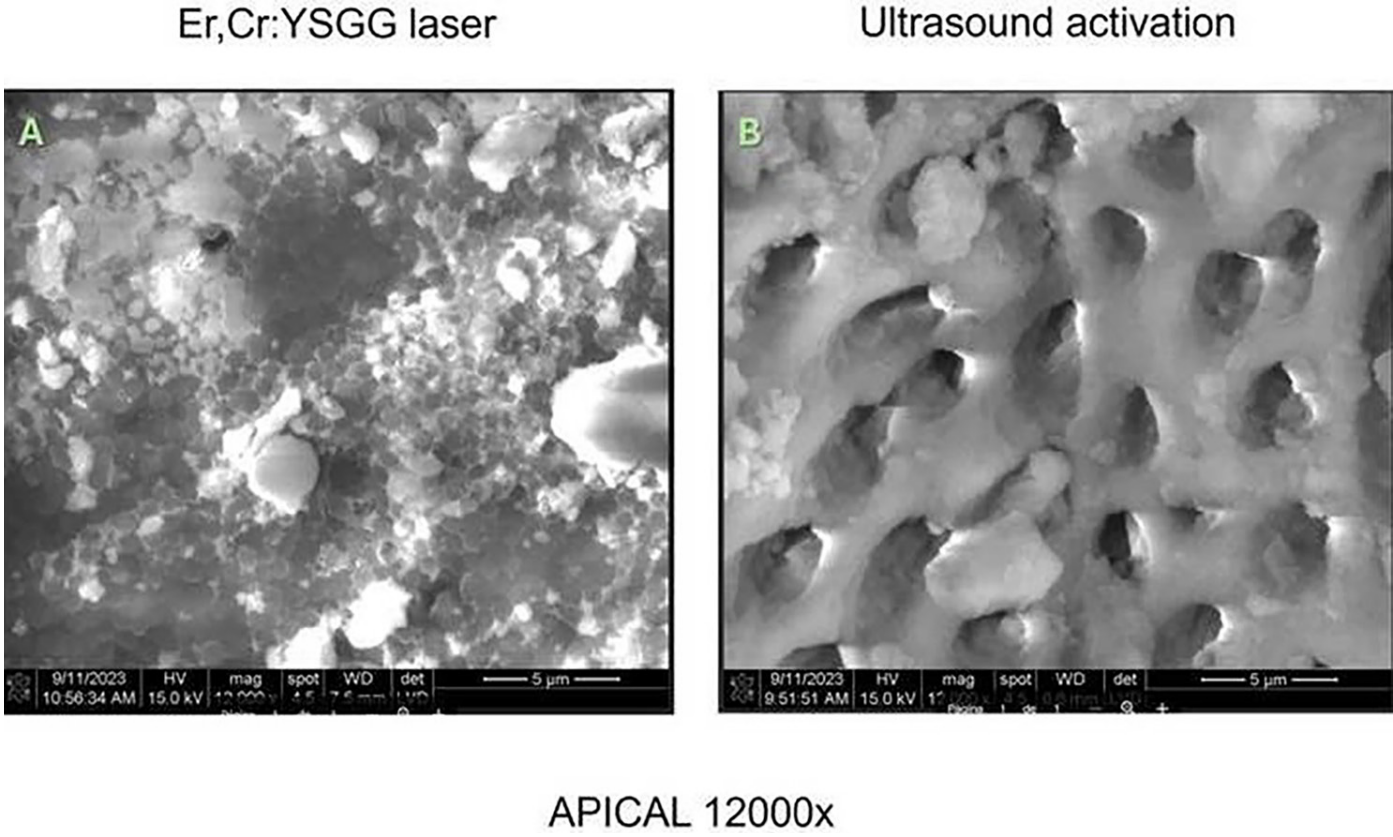
A. A dentinal tubule can be partially seen; dentinal sludge is seen covering the entire dentinal surface with cocci. 4 B. Almost permeable dentinal tubules are seen with a very light layer of dentinal sludge.

**Figure 5:**
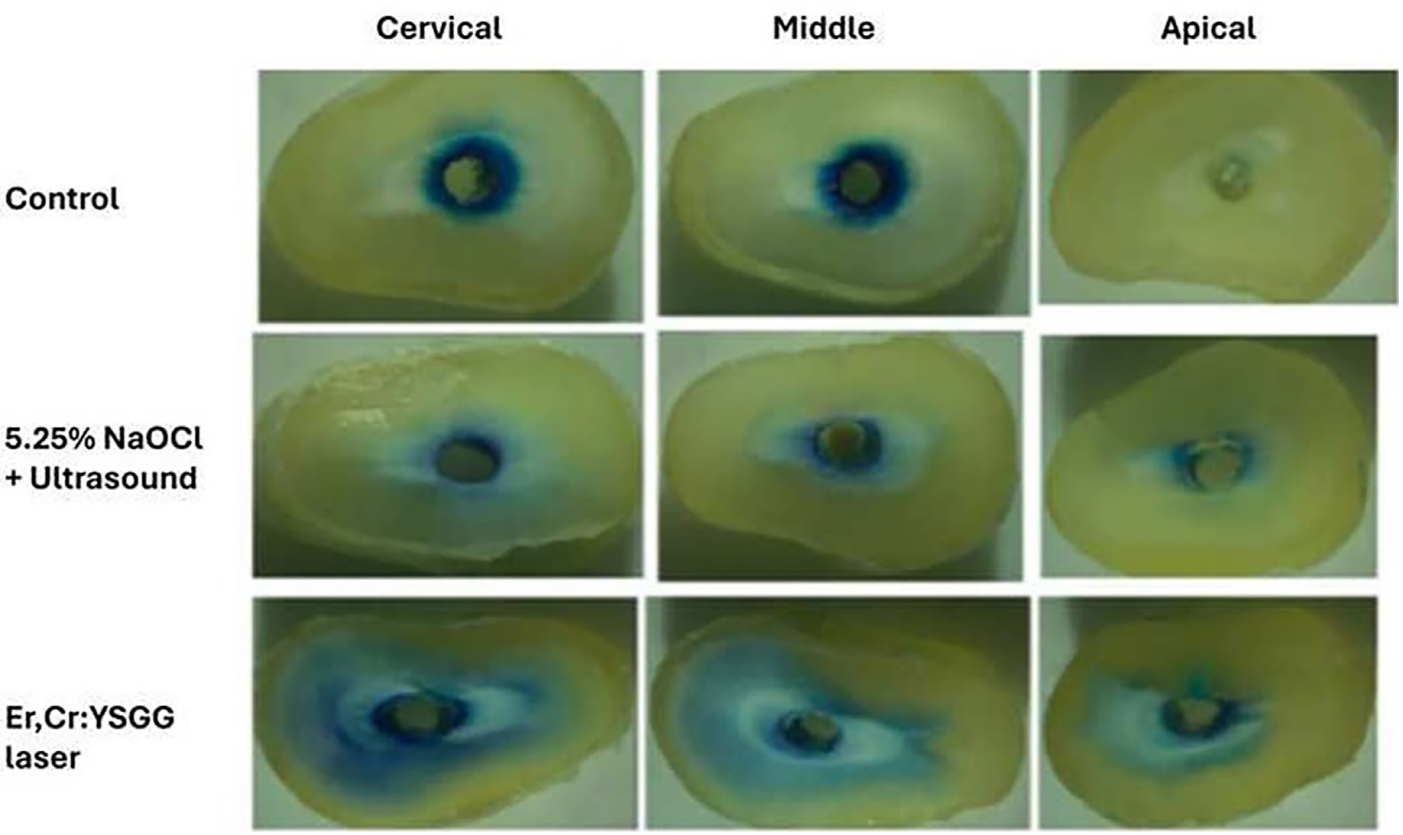
Methylene blue penetration in the cervical, middle and apical third according to the treatment used.

No significant differences were observed in mean bacterial growth before and after treatment in the Er, Cr laser group (*p* > .05). However, significant differences were observed in the US + 5.25% NaOCl group (*p* > .05) (Fig. 1). SEM images indicated more effective smear layer removal in the Er, Cr laser-treated group, particularly in the apical third (Figs. 2,3,4). Additionally, greater methylene blue penetration was observed in the Er, Cr group (Fig. 5).

## Discussion

This study investigated the efficacy of passive ultrasound and Er, Cr laser irrigation techniques on biofilm removal and dentinal tubule permeability. Results indicated no significant bacterial reduction with Er, Cr laser, whereas significant reductions were observed with ultrasound-activated NaOCl. SEM analysis revealed more effective smear layer removal in the Er, Cr laser-treated group.

These results are similar to those reported by Betancourt *et al*. (10), Chauhan *et al*. (11), and Keskin *et al*. (12), who compared the effectiveness of various laser systems, including Er,Cr. They reported that the Er,Cr laser did not significantly reduce bacteria, while ultrasound-activated NaOCl was effective in bacterial reduction. Additionally, the Er,Cr laser was found to be more effective in smear layer removal.

Additionally, *Enterococcus faecalis* which is a primary pathogen associated with endodontic treatment failure (13-14), has been utilized to create biofilms simulating endodontic infection conditions (15). Chemomechanical preparation is essential for cleaning, disinfecting, and shaping the root canal, aiming to eliminate infected pulp tissue, dentin remains, and viable microorganisms (13). Sodium hypochlorite (NaOCl) has been the most used endodontic irrigant since the early 20th century (16), despite its high toxicity potential (17).

The higher antibacterial effect of NaOCl activated by ultrasound can be attributed to the energy transmission into the root canal, which induces cavitation and acoustic streaming (18). In recent years, various laser types have been employed in endodontic treatment (19). Erbium lasers, effective in root canal preparation, can also remove the smear layer and disinfect root canal walls (20). However, their clinical adoption is limited by high costs, potential thermal damage, and technique sensitivity (21). The Er, Cr laser generates explosive vapor bubbles, creating hydrodynamic shear forces that remove debris and disrupt bacterial biofilms (22). Complementary techniques, such as ultrasonic irrigation, should be analyzed in enhancing cleaning and disinfection of root canals (23).

This study demonstrates several innovative strengths in the field of root canal disinfection. Notably, it directly compares the Er,Cr laser with ultrasonic irrigation using 5.25% NaOCl, representing two advanced methods for endodontic treatment. The use of the Er,Cr laser is particularly noteworthy due to its high absorption in saline solution and its affinity for hydroxyapatite, which enhances its ability to remove the smear layer. Additionally, the study employs detailed techniques such as scanning electron microscopy (SEM) to assess dentinal tubule permeability, integrating traditional microbiological methods with a comprehensive analysis of cleaning efficacy. These combined methodologies provide a thorough and novel perspective on the effectiveness of disinfection techniques in endodontic treatment.

Despite its strengths, the study has some limitations. The sample, while carefully selected, is relatively small, which may limit the generalizability of the findings. Additionally, the absence of a formal power analysis prior to data collection could affect the statistical robustness of the results. The study is also limited to an *in vitro* evaluation, meaning the results may not fully reflect clinical efficacy under real treatment conditions. Lastly, while the Er,Cr laser was used with specific parameters, variability in technique and equipment calibration could influence the consistency of the results.

## Conclusions

While the Er, Cr laser did not significantly reduce bacterial counts, the ultrasound-activated NaOCl solution achieved a substantial reduction in bacterial presence. Additionally, scanning electron microscopy (SEM) showed that the Er, Cr laser was more effective in removing the smear layer compared to other methods. Overall, these results suggest that while ultrasound-activated NaOCl is superior in reducing bacterial load, the Er, Cr laser may offer advantages in smear layer removal, which could enhance overall endodontic treatment outcomes. Further clinical studies are required to validate these findings and evaluate the long-term success of these irrigation techniques. Future studies could incorporate a more rigorous power analysis to enhance the statistical robustness of the findings.

## Data Availability

The datasets used and/or analyzed during the current study are available from the corresponding author.
